# Co-developing an integrated primary care workforce planning approach at a regional level: overarching framework and guiding principles

**DOI:** 10.1186/s12960-021-00578-z

**Published:** 2021-07-21

**Authors:** Ivy Lynn Bourgeault, Caroline Chamberland-Rowe, Sarah Simkin

**Affiliations:** University of Ottawa and Canadian Health Workforce Network, Ottawa, Canada

**Keywords:** Integrated health workforce planning, Primary care, Regional planning, Multi-professional, Population health needs, Conceptual framework

## Abstract

Health workforce planning provides a crucial evidence-base for decision-makers in the development and deployment of a fit-for-purpose workforce. Although less common, health workforce planning at the regional level helps to ground planning in the unique realities of local health systems. This commentary provides an overview of the process by which an integrated primary healthcare workforce planning toolkit was co-developed by university-based researchers with the Canadian Health Workforce Network and partners within a major urban regional health authority. The co-development process was guided by a conceptual framework emphasizing the key principles of sound health workforce planning: that it (1) be informed by evidence both quantitative and qualitative in nature; (2) be driven by population health needs and achieve population, worker and system outcomes; (3) recognize that deployment is geographically based and interprofessionally bound within a complex adaptive system; and (4) be embedded in a cyclical process of aligning evolving population health needs and workforce capacity.

Health workforce planning (HWP) provides a crucial evidence-base for decision-makers in the development and deployment of a fit-for-purpose workforce capable of addressing population health needs and achieving desired population health outcomes. As Hall and Mejia [1] described in their foundational piece, it is a:

“process of estimating the number of persons and the kind of knowledge, skills, and attitudes they need to achieve predetermined health targets and ultimately health status objectives.[…] This planning must be a continuing and not a sporadic process, and it requires continuous monitoring and evaluation” (p.18).

That is, HWP ought to be an ongoing process informing decisions that impact the health and wellbeing of populations, the employment potential and career paths of health workers, and the return on investment of public institutions intent on making optimal use of finite resources. The rigour of methods employed and the quality of input data into HWP activities are therefore of critical importance. Ideally, it should be a multifaceted, cyclical process for continuous improvement.

Given the considerable economic and human costs involved in the provision of appropriate healthcare—costs which are largely attributed to the health workforce—poor health workforce planning is almost as detrimental as the absence of planning [2, 3]. In Canada, for example, the direct costs of the health workforce amounted to $175 billion in 2019, or nearly 8% of Canada’s total GDP.^1^

In this commentary, we describe the overarching framework and key principles that guided the development of an integrated primary care HWP toolkit co-developed in partnership with regional healthcare decision-makers. Recognizing the multifaceted nature of HWP, reflective of the complex and adaptive nature of the health workforce system [4], the toolkit that emerged from this partnership is a collection of fit-for-purpose qualitative, descriptive and quantitative planning processes that together guide and support a regional health authority in conducting high-quality HWP activities.

## Shifting health workforce planning to a regional level

Health workforce planning is typically undertaken at national or subnational (provincial/state) levels due, in part, to the location of interested stakeholders and data custodians within systems, and the level at which healthcare decisions are made. In a federated system such as Canada, but also in the US and Australia, most of the policy levers that can be used to adjust health workforce supply, distribution and mix are at the provincial, state or territorial level, and as such, planning (where it exists) typically occurs at this level.

Planning capability at regional levels of geography, however, is increasingly necessary to ensure that the healthcare needs of defined populations can be met by locally available health workers. Indeed, leading HWP practices encourage approaches to be grounded in the unique realities of local health systems. Such approaches can inform the development of targeted health workforce recruitment, deployment and management strategies to address persistent deficiencies in workforce supply, distribution and mix.

Having identified health workforce planning as an essential input to the implementation of their broader comprehensive Primary Care Strategy, the Toronto Region (formerly the Toronto Central Local Health Integration Network), a regional health authority in a major urban centre in Canada, sought guidance from a recognized source of HWP expertise. The result was a partnership with researchers at the Canadian Health Workforce Network (CHWN) with the goal of co-developing an evidence-informed, fit-for-purpose approach to integrated primary care workforce planning. Recognizing that HWP is an iterative process that leverages both quantitative workforce and population health data and qualitative workforce intelligence elicited from key stakeholders, a multifaceted toolkit approach was recommended as most appropriate.

Monthly teleconferences and several in-person meetings between CHWN researchers and the Health Analytics team for the Toronto Region allowed for the integration of local input throughout the toolkit development process. The Toronto Region’s Primary Care Regional Council, composed of clinician leaders from each of the health authority’s subregions, was also consulted to gather qualitative intelligence during the toolkit development process. The Health Analytics team and Regional Council provided input into the policies and strategies framing their vision for integrated primary care within the region, their planning objectives, the research and policy questions they were seeking to address, and the key workforce challenges they were facing. Each of these guided the development of the different analytic tools to be included in the toolkit. This approach not only helped to build HWP capacity amongst the Health Analytics team, but also trust in the ensuing approach from clinical leaders and other key local stakeholders.

**Fig. 1:**
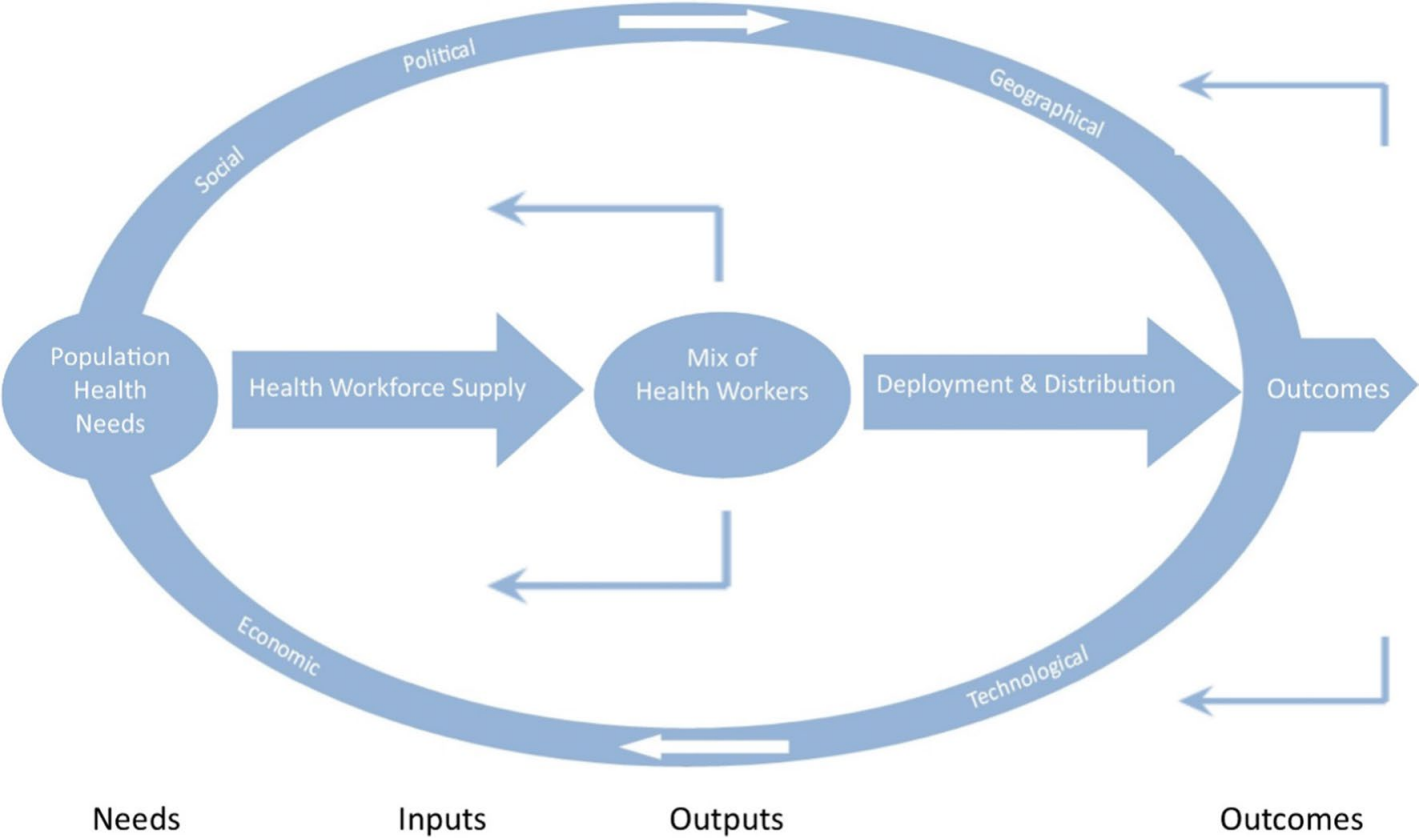
A health workforce systems framework

This integrated approach, described in further detail in the papers led by Chamberland-Rowe [5] and Simkin [6], was informed by a HWP framework, depicted in a simplified form in Fig. 1 [7], which expands upon an earlier model informing a pan-Canadian Health Human Resources Strategy [8]. Briefly, this framework outlines an iterative process by which population health needs drive health workforce planning, the output being an appropriate mix of health workers, deployed and distributed across specific geographies in a manner that ultimately produces positive health, provider and system outcomes. The dynamic process of HWP must recognize the broader social, political, geographic, economic and technological context within which it is situated.^2^

Although this framework was developed for a pan-Canadian approach to health workforce planning, as a guiding framework it emphasizes some of the key principles of high-quality HWP that can be incorporated at a regional level. Specifically, it emphasizes that HWP should:

- be driven by the health and healthcare needs of specific populations;
- acknowledge that health workforce deployment is both interprofessionally based and geographically circumscribed, and is situated within broader social, political and economic contexts; and
- be informed by available evidence across each of these elements, utilizing the highest quality data to best align inputs, activities and outputs.

Indeed, further to the second point noted above, regional planners are able to make decisions related to workforce deployment that directly affect access to appropriate and acceptable care close to home. For instance, HWP tools can be used to determine the optimal shape, size and location of services when planning for new housing development (which often does not consider location of health services).

## Review of leading practices in health workforce planning

The first step in providing guidance to support our Toronto Region partners was to identify leading practices in HWP employed in the Canadian and broader international contexts. Paper 1 [5] in this two-part series outlines the methods and findings from this review and describes the qualitative tools curated for inclusion into the toolkit. High-income countries with similarly federated health system structures and current WHO guidance were a specific focus of the review.

**Fig. 2:**
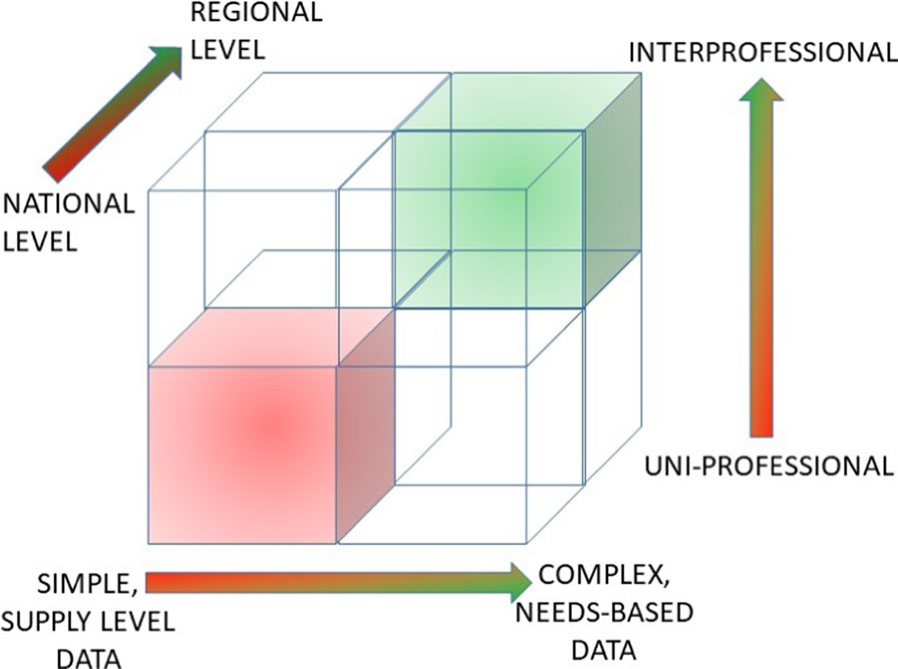
Key dimensions of HWP models

We wanted to move away from typical approaches, at least in the Canadian context, which focus on one profession and employ only simple health workforce supply data (i.e. head counts) at a national level (Fig. 2, bottom left cube at the front, shaded in red). Instead, we prioritized methods which took a more complex, interprofessional, and needs-based approach, which could be deployed at a regional level (Fig. 2, top right cube at the back, shaded in green).

To better reflect the complexity of planning within a complex adaptive health workforce system [4], quantitative modelling processes should be informed by qualitative scenario-based processes. Thus, the toolkit incorporates a mixed-methods approach to better enable regional planners to capture and integrate a diverse range of available data sources—both quantitative and qualitative—into their planning exercises and policy-making processes.

Our approach diverges explicitly from the creation of obscure, black-box approaches developed by consultants at arms-length, projecting out 10 to 25 years with little practical applicability, ease of use, or adaptability. Whilst long-term planning is of value given the time lag involved in producing highly skilled health professionals [9], these planning horizons introduce a high degree of uncertainty into projections. This uncertainty is particularly problematic in complex adaptive health systems that depend so highly on human capital and need to be responsive to emerging drivers of change. Long-term projections, that reflect a range of possible futures through scenario analyses, can be useful in establishing a long-term vision for health workforce management. Approaches can also benefit from shorter planning horizons that feed into longer-term plans, that are regularly updated to account for new data and emerging trends [10].

Leading practices in HWP increasingly acknowledge that it should involve flexible and iterative planning processes that allow planners to incrementally refine estimates [10–14]. In these iterative processes, continuous monitoring and evaluation can also be used to assess the validity of assumptions, test and refine the accuracy of model estimates, and gauge the effectiveness of chosen policy levers [10, 14]. Because of the adaptive nature of health workforce inflows, outflows and practice patterns (i.e. workforce trends), an ongoing, iterative and interactive process was recommended, enabling shorter-term projections (e.g., one to three years) and regular course correction to promote more stable alignment of health workforce supply with population health needs. Embedding this cyclical approach into a culture of planning within the partner organization encouraged model co-development and co-ownership and required capacity-building to support sustainability and buy-in from stakeholders. This became an additional principle of HWP reflected in our toolkit.

## Scanning available population and health workforce data

The second paper [6] in this two-part series describes the development of a modular quantitative model that followed the principles of population needs-based planning incorporating both population health and health workforce components. The variables included in this model were informed by: (1) the availability of high-quality data to our regional partners; and (2) a focus on the policy levers that are (and are not) within regional planners’ sphere of influence. Regional planners, for example, do not have education and training policy levers within their remit, thus these were not included in the toolkit. Relevant modules could be integrated into the toolkit for stakeholders who have discretion over this policy lever.

The integrated qualitative scenario-based process and quantitative model benefited from a number of advances in data and analytics at the municipal level of the City of Toronto, at the provincial level of Ontario, and national level through the Canadian Institute for Health Information. As a result of an open data initiative and a partnership with a provincial research institute and data steward, the Toronto Region has a remarkable number of high-quality datasets available to describe the demographic and health characteristics of their population at the neighbourhood level. HWP at this level does, however, benefit from investments in standardized health workforce data infrastructure at the provincial/state or national level, recognizing that workforce and patient mobility allow service delivery and care seeking to cut across regional boundaries.

At the provincial level, the province of Ontario, within which Toronto is situated, developed the Health Professions Database through an amendment to the Regulated Health Professions Act in 2007, mandating that all health professional regulatory authorities provide data to the Ontario Ministry of Health following a 59-element minimum standard. Although the Health Professions Database is not yet readily accessible to regional health workforce planners, the opportunity exists to leverage the data in support of multi-professional health workforce planning.

Finally, at the national level, the Canadian Institute for Health Information has invested resources in the development of a Population Grouping Methodology [15]. A component of this methodology quantifies the connection between population health characteristics and service requirements. The second paper speaks to the importance of the investment into these types of tools that can facilitate detailed, high-quality health workforce planning.

## Operationalizing the model

At the time of writing, the co-authors are continuing to work in partnership with our Toronto-based colleagues to operationalize the model drawing upon the various tools developed and curated. This has included guiding the Health Analytics team to populate the HWP model with available quantitative data, which in turn has been informed by a qualitative, scenario-based process. This implementation phase has made clear the necessity of integrating capacity-building of regional planners into any robust approach to support their ability to undertake cyclical planning processes going forward.

## Conclusion and lessons learned for consideration in other jurisdictions

Some key lessons learned from this initiative for other jurisdictions to consider include acknowledging the importance of …

- using international leading practices to guide the development of local HWP approaches, adapting them to the unique contextual features of local health systems;
- assessing the heuristic value of conceptual frameworks based on practical considerations and adjusting accordingly to ensure the robustness of their applicability;
- establishing an open, transparent, ongoing partnered relationship which not only builds capacity, but also enhances trust and buy-in to the approach, inputs, process and outputs amongst affected community stakeholders; and
- investing in high-quality, readily accessible standardized datasets on the health workforce and population health, not only at the regional level, but also at provincial/state and national levels to enable broader scale-up of health workforce planning approaches and initiatives.

A final word about planning capacity is important to emphasize. There is no surfeit of health workforce planners with the background and competency to undertake these analyses. Often capacity and data infrastructure do not co-exist within the same organization. This poses a barrier to advances in evidence-informed HWP, such as that which we describe in the accompanying papers.

## Data Availability

Data sharing is not applicable to this article as no datasets were generated or analysed.

## Abbreviations

CHWN: Canadian Health Workforce Network
HWP: Health workforce planning

## Footnotes

1 Source: estimated from the National Health Expenditure Data, CIHI, 2019.

2 This has been expanded into the STEEPLED approach, which includes Social, Technological, Economic, Environmental, Political, Legal, Educational and Demographic elements, described more fully in Chamberland-Rowe, Simkin & Bourgeault, [5]).
